# Atheroprotective Effects of *Glycyrrhiza glabra* L.

**DOI:** 10.3390/molecules27154697

**Published:** 2022-07-22

**Authors:** Yuliya V. Markina, Tatiana V. Kirichenko, Alexander M. Markin, Irina Y. Yudina, Antonina V. Starodubova, Igor A. Sobenin, Alexander N. Orekhov

**Affiliations:** 1 Petrovsky National Research Center of Surgery, 119991 Moscow, Russia; t-gorchakova@mail.ru (T.V.K.); alexander.markin.34@gmail.com (A.M.M.); a.h.opexob@gmail.com (A.N.O.); 2 Institute of General Pathology and Pathophysiology, 125315 Moscow, Russia; igor.sobenin@gmail.com; 3 Chazov National Medical Research Center of Cardiology, 121552 Moscow, Russia; 4 Peoples’ Friendship University of Russia, 117198 Moscow, Russia; 5 Sechenov First Moscow State Medical University (Sechenov University), 119991 Moscow, Russia; mamaikozlovskaya@gmail.com; 6 Federal Research Centre for Nutrition, Biotechnology and Food Safety, 109240 Moscow, Russia; avs.ion@yandex.ru; 7 Institute for Atherosclerosis Research, Skolkovo Innovative Center, 143025 Moscow, Russia

**Keywords:** licorice, glabridin, atherosclerosis, inflammation, cytokines, cholesterol accumulation

## Abstract

Cardiovascular diseases associated with atherosclerosis are the major cause of death in developed countries. Early prevention and treatment of atherosclerosis are considered to be an important aspect of the therapy of cardiovascular disease. Preparations based on natural products affect the main pathogenetic steps of atherogenesis, and so represent a perspective for the long-term prevention of atherosclerosis development. Numerous experimental and clinical studies have demonstrated the multiple beneficial effects of licorice and its bioactive compounds—anti-inflammatory, anti-cytokine, antioxidant, anti-atherogenic, and anti-platelet action—which allow us to consider licorice as a promising atheroprotective agent. In this review, we summarized the current knowledge on the licorice anti-atherosclerotic mechanisms of action based on the results of experimental studies, including the results of the in vitro study demonstrating licorice effect on the ability of blood serum to reduce intracellular cholesterol accumulation in cultured macrophages, and presented the results of clinical studies confirming the ameliorating activity of licorice in regard to traditional cardiovascular risk factors as well as the direct anti-atherosclerotic effect of licorice.

## 1. Introduction

Atherosclerosis-based diseases represent one of the key reasons for mortality in developed countries. The timely prevention and treatment of atherosclerosis and cardiovascular disease is an important task of modern medicine and science. We know that atherosclerosis development is a multifactorial process including various pathological mechanisms such as modification of lipoproteins, cholesterol accumulation, endothelial dysfunction, oxidative stress, and inflammation in each step of atherogenesis [1,2,3]. Preparations with multiple anti-atherosclerotic effects are preferable for use as anti-atherosclerotic agents since they can possess more beneficial effects by acting on different mechanisms of atherosclerosis development. Preparations based on natural products have a great potential for the early prevention of subclinical atherosclerosis since they usually have pleiotropic mechanisms of action. Most preparations based on medicinal plants can be prescribed for long-term use in people free of clinical manifestations of cardiovascular disease (CVD) but who have an increased risk of progressive atherosclerosis, and in the case of prevention of subclinical atherosclerosis, the risk of serious side effects of natural preparations is significantly lower than with the use of medicines [4,5]. Licorice is worth special attention among numerous natural products which possess anti-atherosclerotic potential. This review attempted to determine the status of the PubMed and Scopus databases until May 2022, to highlight current ideas on the anti-atherogenic effects of licorice in experimental and clinical studies. The following keywords were used in different combinations for the literature search: licorice, anti-atherogenic, atherosclerosis, anti-inflammatory, cytokines, cholesterol, and intima-media. In total, 80 articles were selected for the present review.

Licorice, the root or stem of *Glycyrrhiza glabra* L. (Family Fabaceae), has been widely used as a medicinal plant since ancient times. The main ingredient in licorice is glycyrrhizin, which gives it a sweet flavor, so licorice is used as a sweetener and flavoring agent in confectionery, sweets, drinks, and other foods [6]. Licorice has been widely used in traditional Chinese medicine for over 1000 years. The classical theory of traditional Chinese medicine states that “nine out of ten formulas contain licorice” [7]. To date, a huge number of active chemical components have been isolated from licorice: more than 20 triterpenoids and 300 flavonoids. Many studies have shown the wide range of their pharmacological activity. Anti-inflammatory, antitumor, antimicrobial, antiviral, and immunoregulatory effects of licorice have been established, as well as a number of effects that contribute to the protection and treatment of the cardiovascular, respiratory, endocrine, digestive, and nervous systems [8].

Researchers describe multiple anti-atherosclerotic effects of licorice. Thus, glabridin, the most active flavonoid isolated from licorice, possesses pleiotropic activity, affecting various steps of atherogenesis [9]. The antioxidant effect of glabridin inhibits the oxidation of low-density lipoproteins (LDL) [10] and possibly nicotinamide adenine dinucleotide phosphate (NADPH) oxidase, as well as increasing the expression of antioxidant enzymes in macrophages [11]. The anti-inflammatory effect of glabridin decreases the expression of adhesion molecules—intercellular adhesion molecule-1 (ICAM-1), vascular cell adhesion molecule-1 (VCAM-1), and E-selectin in endothelial cells [12], and suppresses the production of inflammatory mediators such as tumor necrosis factor-α (TNF-α), interleukin-1β (IL-1β), nitric oxide (NO) [13]. In addition, glabridin at doses of 15 and 30 mg/kg was described to have a protective effect in a mouse model of cardiotoxicity induced by a single intraperitoneal injection of doxorubicin due to the positive influence on the composition of the intestinal microbiota and changes in the phenotype of colon macrophages towards the M2 phenotype. Glabridin reduced the ratio of the Firmicutes/Bacteroidetes gut dysbiosis markers to a level similar to that in control mice. In addition, it significantly reduced the abundance of the Desulfovibrio genus but increased the abundance of the Helicobacteraceae family and the Lactobacillus genus. Treatment with glabridin resulted in an increase in the butyrate levels in faeces and peripheral blood, as well as a decrease in LPS levels [14]. It is known that the microbiome and its metabolites are closely associated with the development of atherosclerosis [15]. Thus, it can be assumed that one of the possible mechanisms of the anti-atherosclerotic effect of licorice is the modulation of the intestinal microbiota.

Due to its multiple pleiotropic effects, licorice can be used for various pathologies. It has been shown that the active components of licorice, glycyrrhizin and glycyrrhetinic acid, can be effective for the treatment of rheumatoid arthritis, having an anti-inflammatory effect by blocking the cyclooxygenase-2 (COX-2)/thromboxane A2 pathway [16]. Licorice is also effective for the treatment of diabetes and insulin resistance. Thus, glycyrrhizin reduces blood glucose levels, increases blood insulin levels and the number of pancreatic islet cells, and normalizes oxidative stress parameters [17]. In addition, GA exhibits an endothelial protective effect, protecting the endothelium from advanced glycation end-product-induced damage by inhibiting the receptor for advanced glycation end-products/NF-κB pathway, and is thought to be important in the prevention and treatment of diabetic vascular complications [18]. Some compounds isolated from licorice exhibited peroxisome proliferator-activated receptor gamma (PPARγ) ligand-binding activity, decreased adipocyte size, and increased PPARγ mRNA expression levels in white adipose tissue [19,20]. Studies have demonstrated the antitumor activity of licorice; in particular, glycyrrhizin induces the apoptosis of cancer cells through caspase- and mitochondria-dependent pathways [21,22].

All of the above creates a huge potential for the use of licorice in various pathologies. This review will summarize the multiple anti-atherosclerotic effects of licorice, including experimental and clinical studies.

## 2. Anti-Inflammatory Properties of *G. glabra*

It is generally accepted that inflammatory mechanisms play a key role in the development of atherosclerosis [23,24,25]. Pathophysiologically, the inflammatory process at an early stage of atherogenesis includes the following stages: inflammatory activation of endothelial cells, recruitment of monocytes and other leukocytes to the intima of the vessel, the transition of monocytes to tissue macrophages, the uptake of multiple modified atherogenic LDL by macrophages with the formation of foam cells, their secretion of inflammatory cytokines, reactive oxygen species (ROS) and other mediators, which subsequently leads to the death of foam cells (for example, as a result of apoptosis) and the formation of atherosclerotic plaque [26,27,28]. Bioactive components of *G. glabra* may influence the above stages of inflammation associated with atherogenesis, thus exerting an anti-atherosclerotic effect [9].

Compounds isolated from licorice have a pronounced anti-inflammatory effect. Studies show that licorice inhibits toll-like receptor 4 (TLR4) activation, mitogen-activated protein kinase (MAPK) signaling pathway, nuclear factor kappa B (NF-κB) expression, and activator protein (AP)-1 transcriptional activity, which subsequently leads to a decrease in the expression of pro-inflammatory cytokines and chemokines and other inflammatory mediators such as COX-2, inducible nitric oxide synthase (iNOS), TNF-α, IL-6, IL-1β, IL-12, and IL-23 and vascular endothelial growth factor (VEGF) [29,30,31,32,33,34]. It was shown that an intravenous injection of glycyrrhizin inhibited the expression of TLR-4 in BALB/c mice after intraperitoneal administration of LPS as well as a significantly decreased protein content, inflammatory cell counts, TNF-α, IL-1α, IL-6, myeloperoxidase activity, and expressions of COX-2iNOS, and NF-κB [35]. In cultured LPS-stimulated murine macrophages, licoflavanone, a natural compound isolated from *G. glabra* leaf extract, reduced the translocation of NF-κB into the nucleus, thus inhibiting inflammatory signal transduction at the NF-κB pathway level, so significantly decreasing the transcription of iNOS and COX2. In the same study, licoflavanone caused a reduction in MAPKs phosphorylation and activation [36]. The other study on murine macrophages also demonstrated the anti-cytokine effect of *G. glabra* phytoconstituents: isoliquiritigenin significantly decreased LPS-stimulated NO, IL-1β, and IL-6 production, and glabridin significantly inhibited NO and IL-1β [37]. Glabridin has been shown to inhibit the TNF-α-induced expression of ICAM-1, VCAM-1, and E-selectin genes in endothelial cells, which are among the early manifestations of inflammation in atherosclerosis [12]. In addition, it was found that licorice extract significantly increases the production of the anti-inflammatory cytokine IL-10 [38].

Licorice has a pronounced antioxidant activity, which is manifested in the reduction of oxidative stress. Oxidative stress is an imbalance between the production and removal of ROS and nitrogen species and free radicals, causing lipid peroxidation, protein oxidation, etc., which is important in the development of atherosclerosis [39,40]. It has been shown that glabridin reduces the release of superoxide from macrophages and inhibits the cell-mediated oxidation of LDL by inhibiting the activation of NADPH oxidase [41,42]. Glycyrrhizin also has an inhibitory effect on the formation of superoxide and hydroperoxide in macrophages, inhibits the production of prostaglandin E2 by activated macrophages, and suppresses the release of ROS by inflammatory cells [43]. Phenolic components of glabridin provide antioxidant activity due to the absorption of free radicals, the release of hydrogen, the chelation of metal ions, as well as a decrease in mitochondrial lipid peroxidation ability [44,45]. The revealed effects of reducing the activity of iNOS as well as NO allow us to consider licorice to be an effective antioxidant [31,45].

It is known that the expression of paraoxonase 2 (PON2) increases in monocytes during their differentiation into macrophages as a result of NADPH oxidase activation [46]. Therefore, the reduced expression of PON2 observed in hypercholesterolemia may be due to increased macrophage cholesterol rather than increased oxidative stress. Therefore, an increase in PON2 expression can be considered a selective cellular response that contributes to a decrease in oxidative load formation of foam cells [47]. It has also been shown that glabridin increases the expression of PON2 mRNA and protein, which, in turn, reduces oxidative stress and triglyceride accumulation in macrophages and endothelial cells [48].

All of the described above anti-inflammatory and antioxidant effects of licorice indicate a huge potential for its use in the treatment of atherosclerosis since it is considered to be a multifactorial chronic inflammatory disease.

## 3. Anti-Atherogenic Properties of *G. glabra*

Atherosclerosis is accompanied by an excessive accumulation of LDL in the arterial wall. As a result of multiple modifications, LDL is converted into oxLDL, which triggers innate and adaptive immune responses, leading to the progression of inflammation and the development of atherosclerotic complications [49]. It has been shown that licorice significantly reduces the level of cholesterol, triglycerides, and LDL in blood plasma while increasing HDL levels [50], and it also directly affects the atherogenic modifications of LDL, reducing their oxidation [51]. In addition, it was found that licorice compounds can inhibit the activity of acyl-CoA cholesterol acyltransferase (ACAT), the main enzyme involved in the intracellular esterification of free cholesterol by fatty acyl-CoA with the formation of cholesterol ester. The inhibition of ACAT can lead to cholesterol-lowering and anti-atherosclerotic activity by blocking intestinal absorption of dietary cholesterol, inhibiting the hepatic secretion of very-low-density lipoproteins, and preventing the formation of foam cells in vessel walls [52]. The decrease in cholesterol levels may also be associated with the effect of licorice on the activity of enzymes involved in the biosynthesis and catabolism of cholesterol in the liver. It was found that licorice compounds reduced the activity of hydroxymethylglutaryl-CoA synthase and increased the activity of cholesterol-7α-hydroxylase—enzymes regulating these processes [53]. An interesting fact is the synergistic effect of licorice with other plant compounds. For example, The Ger-Gen-Chyn-Lian-Tang, which is a traditional mixture of extracts from four herbs of Chinese medicine, including licorice, is able to reduce cholesterol levels in the atherogenic ApoE−/− mouse model, throughout visibility, due to the activation of adenosine monophosphate-activated protein kinase (AMPK) and peroxisome proliferator-activated receptor α (PPARα), increased the expression of peroxisome proliferator-activated receptor γ (PPARγ), which also provides an anti-atherosclerotic effect [54]. Another study showed that glabridin activates AMPK and subsequently induces the translocation of the insulin-dependent glucose transporter protein GLUT4 in mouse skeletal muscle, thereby preventing hyperglycemia, which is important in diabetes and atherosclerosis [55]. It is known that PPARs can modulate the atherosclerotic process by interacting their direct effects on reducing inflammation and increasing insulin sensitivity with the indirect effects of PPAR activation on glucose and lipid metabolism [56]. Studies have shown that glabridin acts as a PPARγ activator, thereby lowering blood glucose and lipid levels [57]. In addition, some derivatives of glycyrrhetinic acid showed antitumor activity in models using MCF-7, HepG2 cell lines no worse than the classic PPARγ agonist Rosiglitazone, and also showed themselves as low-toxic compounds [58]. Studies have also shown that licorice is a natural inhibitor of 11-beta-hydroxysteroid dehydrogenase type 1, an elevated level of which correlates with the development of obesity, type 2 diabetes mellitus, and insulin resistance [59], which are known to lead to the development of vascular complications, in particular atherosclerosis [40]. Other data also demonstrate the positive effects of licorice (in particular, licorice flavonoid oil containing glabridin on high-fat diet-induced obesity in C57BL/6J mice, manifested in the reduction of hyperinsulinemia, abdominal adipose tissue, visceral fat, as well as body weight. These effects are associated with the suppression of the synthesis of fatty acids and the activation of their catabolism in the liver [60].

Vascular smooth muscle cells (VSMC) have an increased ability to change phenotype, proliferation, and migration, and by penetrating into the intima of the vessel and changing the phenotype, they play an important role in the initiation and progression of atherosclerosis [61,62]. It was found that the licorice flavonoid Isoliquiritigenin significantly reduces the expression of transient receptor potential canonical 5, the main subtype of store-operated channels, localized in VSMCs, thereby inhibiting the proliferation of VSMCs [63]. In addition, the endothelium-independent vasodilatory effect of this compound has been shown [64]. The vasodilating effects of licorice can be realized through endothelium-dependent and endothelium-independent actions. These effects are associated not only with the influence of licorice on the release of NO, but also with a partial blocking of the Inositol trisphosphate receptor (IP3R), which prevents the intracellular release of calcium ions into the cytosol [65]. It was found that the components of licorice can have an estrogen-like effect, modulating vascular damage and atherogenesis. The inhibition of VSMC proliferation is very important for the prevention of atherosclerosis. Studies have shown the estradiol E2-like activity of glabridin, which inhibits VSMC proliferation and stimulates DNA synthesis in endothelial cells when incubated with VSMC and ECV-304 endothelial cells. In addition, in animal studies, glabridin, similar to E2, stimulated creatine kinase (CK), which is a marker of estrogen response in the aorta and left ventricle of the heart [66].

**Figure 1:**
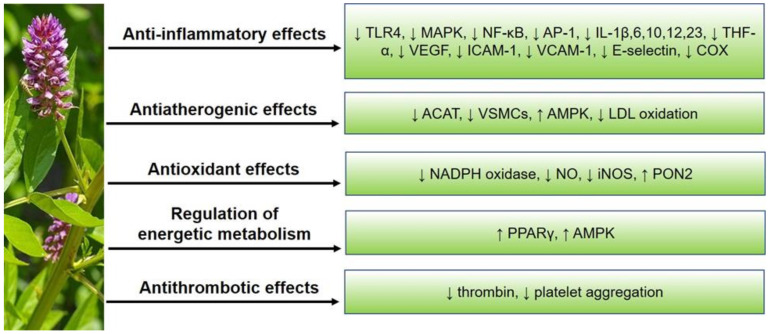
Anti-atherosclerotic effects of *G. glabra*. TLR4, toll-like receptor 4; MAPK, mitogen-activated protein kinase; NF-κB, nuclear factor-κB; AP-1, activator protein-1; IL, interleukins; TNF, tumor necrosis factor; VEGF, vascular endothelial growth factor; ICAM-1, intercellular adhesion molecule-1; VCAM-1, vascular cell adhesion molecule-1; COX, cyclooxygenase; ACAT, acyl-CoA cholesterol acyltransferase; VSMCs, vascular smooth muscle cells; AMPK, adenosine monophosphate-activated protein kinase; LDL, low-density lipoproteins; NADPH oxidase, nicotinamide adenine dinucleotide phosphate oxidase; NO, nitric oxide; iNOS, inducible nitric oxide synthase; PON2, paraoxonase 2; PPARγ, peroxisome proliferator-activated receptor γ.

Among the other positive effects of licorice on atherogenesis, its antithrombotic activity has been studied. The antithrombotic effect of glycyrrhizin is associated with its direct action as an inhibitor of thrombin through exosite I [67]. Licochalcone A, an active ingredient of licorice, was shown to reduce platelet activation due to different mechanisms, in particular, through the inhibition of signaling pathways involved in collagen-mediated platelet aggregation [65]. Licochalcone A also attenuated thrombus formation in the adenosine diphosphate-induced lung thrombosis model of mice and fluorescein sodium-induced platelet thrombus formation in mesenteric microvessels of mice [68]. The major anti-atherosclerotic effects of *G. glabra*. are shown in Figure 1.

### Results of In Vitro Study of the Anti-Atherogenic Effect of Licorice Root Extract

Our recent pilot study was designed to evaluate the effect of *G. glabra* on the atherogenicity of blood serum in an ex vivo model. Blood serum atherogenicity was defined as the ability of serum to induce cholesterol accumulation in the primary culture of human blood-derived monocytes [69]. Natural preparation Lacrinat (INAT-Farma, Moscow, Russia) contained 200 mg of licorice root powder and was used as a source of *G. glabra*. The estimation of serum atherogenicity was performed by measurements of intracellular cholesterol content in cultured cells after incubation with blood serum received at baseline and after 2, 4, and 6 h after Lacrinat administration. Peripheral blood monocytes were isolated from the whole blood of healthy volunteers. Monocytes were isolated by low-speed gradient centrifugation in a lymphocyte separation medium (LSM 1077, Lonza, Switzerland). The isolated monocytes were incubated overnight in Dulbecco Modified Eagle Medium (DMEM, Lonza, Switzerland) with 10% fetal calf serum (HyClone Calf Serum, Thermo Fisher Scientific, Waltham, MA, USA). On the second day, the cells were washed with sterile phosphate-buffered saline (Sigma-Aldrich, St. Louis, MO, USA) and incubated in fresh DMEM containing 10% of the tested serum sample. The control cells were incubated without blood serum. All of the tests were performed in triplicate on the same cell culture for every sample tested. The serum samples obtained from the same participant at baseline and after 2, 4, and 6 h after licorice extract administration were analyzed in a single experiment to allow valid between-sample comparison. After 3.5-h incubation, the cultured cells were washed with phosphate-buffered saline and fixed with the hexane–isopropanol mixture (Sigma-Aldrich, St. Louis, MO, USA). Lipids from the fixed cells were extracted thrice with the hexane-isopropanol mixture, and combined extracts were evaporated under horizontal airflow. The dried extract was dissolved in the detergent mixture containing isopropanol, phosphate-buffered saline, sodium cholate, and Triton X-100 (Sigma-Aldrich, St. Louis, MO, USA). The cholesterol content was determined by an enzymatic assay using commercial kits for total cholesterol measurement (Fluitest CHOL, Analyticon Biotechnologies AG, Lichtenfels, Germany). The measurements were conducted using the colorimetry method (Synergy 4, Bio Tek Instruments, Winooski, VT, USA). The protein content in the delipidated cells was measured by the Lowry method [70]. The cholesterol content was divided by the corresponding protein content and expressed as μg/mg of cell protein.

The pilot study was performed on 15 participants. All of the study participants signed the informed consent form prior to inclusion in the study. The study protocol was approved by the Institute for Atherosclerosis Research Committee on Human Research. All of the participants of the study did not have CVD and other chronic diseases except hypertension and received no regular treatment except for antihypertensive therapy. Ten of fifteen study participants had mild arterial hypertension, so they received hypotensive therapy. These individuals received antihypertensive monotherapy with telmisartan at a dose of up to 40 mg/day (*n* = 8) or losartan at a dose of up to 50 mg/day (*n* = 2). At the time of the experiment, all participants had a normal blood pressure and blood lipid profile. The clinical characteristics of the study participants are presented in Table 1.

The dynamics of intracellular cholesterol accumulation are presented in Table 2. Intracellular cholesterol content in control cells incubated without blood serum was taken at 100%. The intracellular cholesterol content in the experimental cells was expressed in absolute values and as a percentage of the control cells.

The intracellular cholesterol content in the cultured cells increased significantly after the addition of the blood serum obtained before licorice administration, *p* < 0.001, demonstrating the atherogenic potential of blood serum. The ability of blood serum to induce intracellular cholesterol accumulation decreased significantly in 2, 4, and 6 h after intake of a single dose of preparation based on licorice root, *p* < 0.001 at each point. The small number of participants is a strong limitation of the study, but it was demonstrated in the conducted pilot experiment that taking a preparation based on *G. glabra* significantly reduces the ability of blood serum to induce the accumulation of intracellular cholesterol in cultured human blood monocytes/macrophages, that confirms the antiatherogenic potential of licorice and allows considering it as a promising substance for the development of approaches for the prevention and treatment of atherosclerosis.

## 4. Clinical Studies on the Effects of *G. glabra* on Cardiovascular Risk and Atherosclerosis Development

Numerous clinical trials are devoted to studying the effects of preparations based on licorice on different pathologies, in particular, on oral and gastrointestinal diseases, skin disorders, and metabolic disorders [71]. The direct anti-atherosclerotic action of licorice on carotid intima-media thickness (cIMT) progression has been evaluated in a single study. It was demonstrated that 12-months of licorice root extract administration at a dose of 200 mg per day led to a significant reduction of cIMT from 0.92(0.25) to 0.84(0.21), *p* < 0.001 in the experimental group of 51 participants, while in control group of 43 participants cIMT increased from 0.85(0.17) to 0.88(0.18), *p* < 0.001 [72]. In the same study, the lipid-lowering action of licorice was estimated: serum levels of total cholesterol and LDL decreased significantly in the licorice group but did not change in the control group. Interestingly, this study also demonstrates a beneficial effect of licorice on blood pressure, which significantly decreased during 1-year of licorice consumption in comparison with the control group. Another clinical trial confirmed the hypotensive and lipid-lowering effects of licorice in 12 hypercholesterolemic patients. The total cholesterol, LDL and triglycerides blood level as well as systolic blood pressure decreased significantly after 1 month of licorice root extract administration and returned to baseline values after 1 month of follow-up without intervention [73]. A recent systematic review with meta-analysis shows that licorice consumption significantly reduces the body weight and body mass index, which may be beneficial for cardiovascular disease since overweight is considered to be an important cardiovascular risk factor [74]. Licorice is often used in combination with other natural ingredients in complex natural preparations that also possess cardioprotective effects in regard to lipids profile and blood pressure [75]. Moreover, it was shown that bioactive licorice components have a great therapeutic potential in the treatment of diabetes mellitus based on their anti-inflammatory and antioxidant properties [76], which may contribute to the reduction in atherosclerosis progression in diabetic patients.

Despite the fact that the ameliorating effect of licorice preparations on arterial hypertension was described in some clinical studies, numerous studies demonstrate side effects of licorice such as hypertension, metabolic alkalosis, and secondary disorders caused by hypokalemia [11]. Licorice is a source of glycyrrhetinic acid that reduces the activity of the 11β-hydroxysteroid dehydrogenase type 2 isoenzyme, which leads to the activation of the mineralocorticoid receptors by cortisol, so preparations based on licorice may possess corticosteroid-like activity [71]. The complex of adverse symptoms caused by licorice is called pseudohyperaldosteronism, and it has been described in several patients taking licorice preparations for a long time, which led to a disorder requiring intensive care [77]. The meta-analysis, including the results of 18 clinical trials, was performed to estimate the hypertensive effect of licorice. It was demonstrated that in different groups of patients taking licorice, starting with a dose of 100 mg per day (the average daily dose of glycyrrhizic acid in the studies was 377.9 mg), there was a dose-dependent increase in systolic and diastolic blood pressure, but the number of studies is too small, and the design of the included studies is heterogeneous in terms of groups of patients and measured outcomes, that limits the results of meta-analysis [78]. However, these facts may limit the use of licorice in certain groups of patients but do not negate the above positive effects of licorice. Thus, licorice preparations should be used under the physician’s control with caution during pregnancy, in the elderly, and in patients with arterial hypertension.

## 5. Conclusions

Multiple beneficial properties of *G. glabra*, such as anti-inflammatory, anti-cytokine, antioxidant, anti-atherogenic, and anti-platelet effects, demonstrated in in vitro and in vivo models, allow considering preparations based on licorice as a perspective atheroprotective agents. The results of the pilot study presented in this review demonstrate the ability of the preparation based on *G. glabra* to reduce intracellular cholesterol accumulation in ex vivo study, indicating the promising anti-atherogenic potential of licorice. Despite the promising effect of licorice in experimental studies, only a few studies demonstrate the anti-atherosclerotic effects of licorice in clinical trials. However, currently accumulated knowledge about the anti-atherosclerotic effects of *G. glabra* can be a rationale for the wider use of licorice preparations for the prevention and treatment of atherosclerosis. Further clinical studies are needed to verify the use of licorice to ameliorate the traditional CVD risk factors, as well as atherosclerosis regression; studies are required to confirm a direct anti-atherosclerotic effect of licorice, such as reducing the cIMT progression.

## Figures and Tables

**Table 1:** Clinical characteristics of participants of the study on the effect of licorice root powder on serum atherogenicity.

Characteristic	
Age, years	55.1 (4.1)
BMI, kg/m^2^	24.6 (2.7)
Blood pressure, mmHg	126/80 (8/5)
Hypotensive therapy, %	67
Smoking, %	13
CVD, %	0
Total Cholesterol, mg/dL	210.9 (22.9)
TG, mg/dL	114.7 (36.6)
HDL, mg/dL	61.6 (22.0)
LDL, mg/dL	120.8 (24.9)

BMI, body mass index; CVD, cardiovascular disease; TG, triglyceride; HDL, high-density lipoproteins; LDL, low-density lipoproteins.

**Table 2:** Blood serum atherogenicity after licorice root powder administration.

	Control	0 h	2 h	4 h	6 h
Intracellular cholesterol, µg/mg	17.0	28.7 *	21.5 **	19.9 **	20.0 **
	(3.0)	(6.1)	(7.7)	(5.4)	(4.7)
Intracellular cholesterol, % from control	100	170 *	124 **	118 **	117 **
		(29)	(30)	(28)	(15)

*, significant difference at *p* < 0.001 compared to cholesterol content in control cells incubated without tested serum; **, significant changes at *p* < 0.001 compared to cholesterol content in cells incubated with tested serum before licorice root powder preparation administration.

## Data Availability

The data presented in this study are available on request from the corresponding author.
